# High Diversity of Type I Polyketide Genes in *Bacidia rubella* as Revealed by the Comparative Analysis of 23 Lichen Genomes

**DOI:** 10.3390/jof8050449

**Published:** 2022-04-26

**Authors:** Julia V. Gerasimova, Andreas Beck, Silke Werth, Philipp Resl

**Affiliations:** 1 Systematics, Biodiversity and Evolution of Plants, LMU Munich, 80638 Munich, Germany; beck@snsb.de (A.B.); werth@bio.lmu.de (S.W.); philipp.resl@uni-graz.at (P.R.); 2 Botanische Staatssammlung München, SNSB-BSM, 80638 Munich, Germany; 3 Institute of Biology, University of Graz, 8010 Graz, Austria

**Keywords:** lichen, secondary compounds, comparative genomics, fungi, polyketide synthases (PKS), Type I PKS

## Abstract

Fungi involved in lichen symbioses produce a large array of secondary metabolites that are often diagnostic in the taxonomic delimitation of lichens. The most common lichen secondary metabolites—polyketides—are synthesized by polyketide synthases, particularly by Type I PKS (TI-PKS). Here, we present a comparative genomic analysis of the TI-PKS gene content of 23 lichen-forming fungal genomes from Ascomycota, including the de novo sequenced genome of *Bacidia rubella*. Firstly, we identify a putative atranorin cluster in *B. rubella*. Secondly, we provide an overview of TI-PKS gene diversity in lichen-forming fungi, and the most comprehensive Type I PKS phylogeny of lichen-forming fungi to date, including 624 sequences. We reveal a high number of biosynthetic gene clusters and examine their domain composition in the context of previously characterized genes, confirming that PKS genes outnumber known secondary substances. Moreover, two novel groups of reducing PKSs were identified. Although many PKSs remain without functional assignments, our findings highlight that genes from lichen-forming fungi represent an untapped source of novel polyketide compounds.

## 1. Introduction

Fungi synthesize an extensive array of chemically and functionally diverse natural products, termed secondary metabolites, with roles in defense, self-protection and development [1,2]. Based on their properties and the core enzymes and precursors involved in their biosynthesis, four major groups of fungal secondary metabolites are distinguished: polyketides, non-ribosomal peptides (NRPS), terpenoids and tryptophan derivatives [3]. Most fungal secondary metabolites are encoded by genes located adjacent to each other (i.e., “clustered”) in the genome [2,4].

In lichen-forming fungi, polyketides are the most common class of secondary metabolites [5,6]. Although lichen polyketides can be produced by mycobionts grown axenically under appropriate conditions [7,8,9,10], they are usually formed in intact symbioses [11,12]. Many lichen polyketides are synthesized by Type I polyketide synthases (TI-PKS) [13], the closest structural and functional analogue of which is the mammalian fatty acid synthase [14]. In the minimal configuration, the domain structure of TI-PKSs always includes a ketoacyl synthase (KS), an acyltransferase (AT), and an acyl carrier protein (ACP). These domains are essential for polyketide synthesis [3]. This configuration can be supplemented with domains such as starter unit-ACP transacylase (SAT), ketoreductase (KR), dehydratase (DH), enoyl reductase (ER), methyltransferase (CMeT), and thioesterase (TE) [3].

The presence of optional domains has led to the classification of fungal PKSs into three subgroups based on their ability to perform redox reactions. The first subgroup comprises non-reducing (NR) PKSs, which lack reductive domains and mainly produce aromatic polyketides [13,15]. The second subgroup contains partially reducing (PR) PKSs, typically having a single KR or a KR and DH domain. Finally, reducing (R) PKSs contain a complete set of reductive domains, viz. KR, DH, and ER. All three subgroups can be found in lichen-forming fungi. Previous work suggests various ecological roles of polyketides in lichens ranging from light-screening and chemical weathering to allelopathic effects and herbivore defense [3,6,16,17]. More broadly, secondary metabolite profiles including polyketides are often characteristic of taxonomic groups and are thus extensively used to identify lichens.

The first PKS gene from a lichen-forming fungus was cloned and analyzed by Armaleo et al. in 2011 [18], and in recent years secondary metabolite research has benefited from genome mining approaches in bacteria, fungi, and plants, uncovering hidden diversity (e.g., [19,20,21]). The fact that genes encoding natural product biosynthetic pathways are often clustered in the genome [2] facilitates the identification of biosynthetic gene clusters (BGCs) in whole genome sequences. In many cases, the chemical structure of their products can be predicted from the biosynthetic logic of enzymes encoded in a BGC and their similarity to known counterparts [22,23,24,25]. Despite this progress, the biosynthetic genes for most secondary metabolites of lichen-forming fungi remain uncharacterized. Observations that lichen-forming fungi contain more biosynthetic genes than characterized chemical compounds also complicates clear gene assignments to particular metabolites. Consequently, previous studies have focused only on a few well-known compounds, e.g., usnic acid, grayanic acid, and atranorin (e.g., [18,25,26,27,28,29]). However, the increasing availability of lichen-forming fungal genomes provides a largely untapped resource for identifying additional biosynthetic genes [30]. 

Here, we investigate the diversity of BGCs in the de novo sequenced *Bacidia rubella* (Hoffm.) A. Massal. (Ramalinaceae) genome within a two-level comparative genomic framework. We aim to identify genes involved in the biosynthesis of atranorin, a secondary metabolite present in many lichens including *B. rubella.* To achieve this, we examine the secondary metabolite biosynthetic potential of *B. rubella* by comparing its genome to that of Ramalinaceae species: *Bacidia gigantensis*, *Ramalina intermedia* and *R. peruviana*. The secondary metabolites of these four closely-related species are known, and the chemical substance profiles overlap. This facilitates comparison of the presence, absence and structure of PKS genes, and enables the evaluation of BGCs for previously characterized genes, including those encoding for atranorin biosynthesis. We then extend this comparative approach to include an additional nineteen, publicly available fungal genomes from the Lecanoromycetes. Most of these genomes were obtained from pure cultures, with high genome completeness and little contamination. By employing an in-silico approach combined with phylogenetic reconstructions of previously characterized sequences, we gain insight into the putative functions of TI-PKS BGCs in lichen-forming fungi. Our comparative genomic results are congruent with previous work, indicating a high diversity of BGCs in lichen-forming fungi that extends beyond what can be observed from chemical profiles (e.g., [9,25]). This sheds new light on the potential of lichen–forming fungi to produce different secondary metabolites, with direct relevance for natural product research and production.

## 2. Materials and Methods

### 2.1. In Vitro Cultivation of the Bacidia rubella Mycobiont

The lichen-forming fungus *Bacidia rubella* was axenically cultivated from a specimen collected from Germany (Bavaria, Lkr. Neuburg-Schrobenhausen, Markt Rennertshofen, south-east of Bertoldsheim, Naturwaldreservat “Mooser Schütt”; mixed forest, on bark of the trunk of *Fraxinus* sp., ca. 1.0 m above the ground, ca. 400 m asl.; M-0307710) in April 2019. The mycobiont culture was obtained from a multispore discharge of a single apothecium of *B. rubella* following the method of Yoshimura et al. [31]. Briefly, young and middle-aged apothecia were detached from the thallus and soaked in sterile water for about an hour. Then, they were fixed to the top of a Petri dish lid using petroleum jelly while keeping the lid slightly open to let the apothecia dry slowly. We used Bold’s Basal Media (BBM) with doubled nitrate (25 g/L) as an initial substrate. Upon germination, the spores were transferred to a malt-yeast extract medium (Lichen medium) [32]. The mycobiont cultures were stored in a growth chamber (Wachstumsschrank Binder KBWF 720; Binder GmbH, Tuttlingen) at 16 °C and 60% relative humidity. They were subcultured every two to three months until a sufficient biomass for genomic analysis was obtained (ca. one year).

### 2.2. DNA Isolation and Sequencing

To obtain concentrated high-molecular-weight genomic DNA, about 1 cm^2^ of mycelium was taken and ground in liquid nitrogen with a pre-cooled pistil. Genomic DNA was isolated using the MagAttract HMW DNA Kit following the manufacturer’s protocol and subsequent purification steps with magnetic beads (HMW genomic DNA from fresh or frozen tissue protocol), resulting in a total yield of 1 µg. The final concentration was 9.53 ng/µL, measured with a NanoDrop 1000 spectrophotometer (Peqlab Biotechnologie, GmbH, Erlangen, Germany) and Qubit 4 Fluorometer (ThermoFisher Scientific, Waltham, MA, USA) using 1X dsDNA HS Assay Kits. DNA extraction, PCR amplification and Sanger sequencing (using nrITS primers) were performed to evaluate possible contamination and confirm the cultures’ identities. First, a paired-end library was constructed using Illumina DNA Prep (earlier known as Nextera DNA Flex Library Prep) and sequenced on NovaSeq 6000 (NovaSeq SP 150 bp Paired-end Flow Cell, Illumina) at the Biomedical Sequencing Facility (BSF, Vienna, Austria). A total concentration of 0.2 µg was used for Illumina library preparation. To supplement the Illumina data, we prepared several Oxford Nanopore libraries using the SQK-LSK109 kit and sequenced them on a MinION sequencer using R9.4.1 flow cells (altogether, four runs were conducted). The total yield of DNA was in the range of 0.12–0.31 µg.

### 2.3. Data Generation and Initial Read-Quality Assessment

To obtain a high-quality genome, we used a hybrid assembly approach using Illumina short-reads and Oxford Nanopore long reads. In total, we produced 17 Gbp of raw paired-end Illumina reads with 500× coverage. Raw data inspection with FastQC v0.11.7 [33] indicated high-quality reads (quality score (Q) above 34) and no excessive adapter contamination or read duplication. Basecalling of Nanopore raw signals was performed using Flappie v2.1.3 (git commit 4de542f; https://github.com/nanoporetech/flappie) into a total of 22 Gbp of raw sequences up to 94.5 Kb of read length.

### 2.4. Genome Completeness and Quality Assessment

The paired-end Illumina reads and unpaired nanopore reads were assembled using hybridSPAdes [34] with k-mer sizes of 33, 55, 77 and 127. To examine the assembly for potential non-target contigs, de novo assembly was subjected to BLASTX using DIAMOND [35] against a custom database comprising the protein sets of the NCBI nr database (downloaded in July 2018). The results of this DIAMOND search were used as input for blobtools v1.1.1 [36]. The final results showed the absence of foreign contigs; therefore, further filtering was unnecessary. The quality of the assembly and genome statistics were assessed using QUAST v5.0.2 [37]. The completeness of the genomes was assessed using all single-copy BUSCO genes of the ascomycota_odb9 set (part of the phylociraptor pipeline; git commit 4cfd3c4; accessed on 2 July 2021; see below).

### 2.5. Dataset Construction

The dataset consists of the de novo sequenced genome of *B. rubella* and twenty-two additional representative genomes of Lecanoromycetes, the largest radiation of lichen-forming fungi. We included in our study twenty-three genomes obtained from pure fungal cultures with the exception of *B. gigantensis* and *R. intermedia*, which were obtained by sequencing the whole thallus (Table 1). The dataset aims to identify known and possible unknown BGCs placing them together with previously characterized PKSs from other fungi and bacteria. The genomes were downloaded using phylociraptor, which automatically downloads genomes from NCBI and combines them with additionally specified genomes provided by the user [38]. We kept the original taxon names from NCBI for convenience, even though the current taxonomic status might differ.

### 2.6. Genome Annotation

The publicly available genomes were sequenced with different sequencing technologies at different times and are thus of varying quality. To make annotations comparable, we performed ab initio gene calling and functional annotations for all of them. The downloaded genomes were analyzed using the smsi-funannotate (https://github.com/reslp/smsi-funannotate; git commit 398a144; accessed on 4 June 2021) pipeline based on funannotate v.1.8.7 [39]. First, we removed duplicated identical contigs (funannotate clean) in each assembly. To avoid long contig/scaffold names, we sorted our assembly by contig length and then renamed the fasta headers (funannotate sort). Afterwards, we made a soft repeat masking of the assembly using tantan (funannotate mask) [40]. The following steps included gene prediction using the gene-callers Augustus v3.3.2 [41], snap [42], GlimmerHMM v3.0.4 [43] and Genemark ES v4.68 [44]. For Augustus, we used *Aspergillus nidulans* as a pre-trained species. All the pipelines were run on the cluster of the University of Graz and our in-house Linux Server at LMU Munich. The genome statistics are summarized in Table 4 (See Section 3.2. Biosynthetic Gene Composition in Twenty-Three Annotated Fungal Genomes). The output files of the newly annotated genomes from NCBI and JGI used in this study are available on figshare (http://doi.org/10.6084/m9.figshare.19487837).

### 2.7. Annotation of Biosynthetic Gene Clusters

The identification, annotation and analysis of secondary metabolite BGCs in the studied fungal genome sequences were performed with antiSMASH v6.0 as a part of the smsi-funannotate pipeline and thus all BGC numbers are referred to antiSMASH prediction. We chose all BGCs from the annotation results that contained orthologous core TI-PKSs for our comparative genomic and phylogenetic analyses. The GBK output files with annotated BGCs and predicted genes were visualized on the antiSMASH webserver (fungal version; accessed: November 2021) [45].

### 2.8. Identification of Atranorin Biosynthetic Gene Cluster and Phylogeny

To identify atranorin candidate genes, we downloaded sequences from PKS16 of *Cladonia grayi* (GenBank: ADM79459; 2089 aa) and PKS23 of *Stereocaulon alpinum* (GenBank: QXF68953; 2500 aa). Both PKSs were previously reported as possible candidates involved in the biosynthesis of atranorin (see Section 3.1.1 Atranorin in Section 3 Results and Discussion for details). As atranorin synthesis involves an oxidation, the cluster must contain a cytochrome P450 as well as additional genes, *O*-methyltransferase (OMT), and transporter gene [29]. The presence of these genes was verified by our annotation results, showing OMT (PF08241), cytochrome P450 (PF00067), and transporter gene (PF00400) present in the putative atranorin BGC in *B. rubella*. The gene arrows plot, comparing two atranorin clusters in *Bacidia rubella* and *Cladonia rangiferina*, was drawn using the gggenes v0.4.1 R package (https://github.com/wilkox/gggenes/; accessed on 20 April 2022). 

We used BLASTP v2.9.0+ [46] to detect the orthologs of those characterized sequences in all PKS sequences we identified on the twenty-three studied genomes. We filtered BLAST output to retain sequences with a minimum identity of 30% over the alignment length and a minimum query coverage of 50%, sorted for the highest bit score and lowest e-value. Additionally, we used the best blast subject hits with the highest percent similarity to the query gene. The best hits from the blast results (>30%) were selected for the PKS23 alignment. The taxon selection was confirmed with the clade from the large TI-PKS phylogenetic tree.

We aligned the 29 identified amino acid sequences using MAFFT v7.480 [47] and calculated the maximum likelihood using IQ-TREE v2.1.4 [48] with 1000 ultrafast bootstrap replicates, after selecting the best-fitting substitution model (LG+G8+F) with ModelFinder [49]. The final alignment comprised 29 sequences from 16 taxa, with 2320 amino acid sites, 2071 distinct patterns, 1756 parsimony-informative, 221 singleton sites, and 343 constant sites.

### 2.9. Topology Test to Confirm Atranorin Biosynthetic Genes

**Figure 1:**
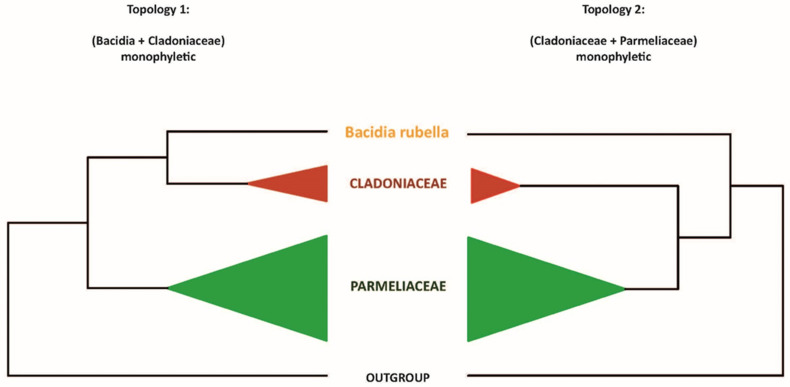
Two alternative topologies used for comparison in the topology test. Topology 1, recovering *Bacidia* + Cladoniaceae as monophyletic; Topology 2, recovering Cladoniaceae + Parmeliaceae as monophyletic.

Our maximum-likelihood phylogeny recovered the *B. rubella* (Ramalinaceae) atranorin gene (bacrubpred_000804) as sister to a clade comprised of *Cladonia rangiferina* and *S. alpinum* (claranpred_005882 and QXF68953, respectively), which both belong to Cladoniaceae. Miadlikowska et al. [50] recovered Parmeliaceae as the closest relative of Cladoniaceae, with a clade of these two families being sister to Ramalinaceae. Thus, we tested if the monophyly of a clade comprising *Bacidia* + Cladoniaceae is significantly supported against the expected phylogenetic relationship comprising Parmeliaceae + Cladoniaceae. For this, we compared (1) an unconstrained ML tree to recover *Bacidia* + Cladoniaceae as monophyletic, and (2) a constrained tree with *Bacidia* sister to Cladoniaceae + Parmeliaceae (Figure 1).

For the topology test, we used PKS23 sequences in the strict sense, i.e., from the species known as atranorin produces with *Gyalolechia flavorubescens* and *Xanthoria elegans* as outgroups. The constrained tree can be multifurcating and need not contain all species; therefore, finally, we shortened our tree to the taxa we wanted to test. First, we performed a constrained search for both topologies using the LG model for the amino acid dataset. Then, we concatenated both trees and implemented tree topology tests, based on the Kishino-Hasegawa test [51], Shimodaira–Hasegawa test [52], Expected Likelihood Weight [53], and approximately unbiased (AU) test [54] using IQ-TREE (-m LG -z concatenated_trees.treels -n 0 -zb 10,000 -au). We considered tree topology to be unlikely if its test *p*-value was <0.05 (marked with a “-” sign). The trees were visualized and examined using FigTree 1.3.1 [55].

### 2.10. Identification of Orthologues and Orthogroups with BUSCO and OrthoFinder

Orthofinder uses a hybrid approach based on sequence similarity estimated by a BLAST all-vs-all search with DIAMOND and subsequent reconstruction of gene trees and a species tree to identify (single copy) orthologs and paralogs. To identify orthologues of extracted PKSs, we inferred orthogroups with Orthofinder v2.5.4 [56] for (1) all predicted proteins and (2) all extracted TI-PKSs, independently. The independent runs for two datasets were conducted to check for the concordance of the final results. As the results were not contradictory, we discussed the result of TI-PKS only. The Markov Cluster (MCL) inflation parameter was set up by default (1.5). The trees utilized by Orthofinder were reconstructed using Fasttree v2.1.10 (-m msa, -A muscle, and -S diamond (default)) [57].

### 2.11. Type I Iterative PKS Alignment

We performed a phylogenetic analysis of all TI-PKSs identified in twenty-three studied genomes of Lecanoromycetes, to assess the relationship of non-reducing PKSs (NR-PKS) and reducing PKSs (R-PKS) (including partly reducing PKSs). First, we prepared a list of all identified TI-PKS based on our functional annotations. We retrieved the sequences based on the corresponding gene ID numbers included in the gff3 file of each genome using a custom python script (select_transcript.py: https://github.com/reslp/genomics/blob/master/select_transcripts.py; accessed on 13 October 2021). We included predicted ketoacyl synthase (KS) amino acid alignment reported by Kroken et al. [14] to enhance our dataset. The inclusion of reference PKSs enables us to compare the tree topology proposed in Kroken et al. [14] to our larger sampling of putative PKS genes.

All TI-PKS sequences were combined into a single file and aligned using MAFFT v7.480 [47]. We chose the E-INS-i alignment strategy because it performs better when aligning sequences with several conserved motifs interspersed in long, unalignable regions. We trimmed the alignment using trimAl v.1.4.rev15 [58]. Initial testing of different trimming settings (e.g., -gappyout, -strict, and -automated), showed that parameter combination (-gt 0.70 -resoverlap 0.70 -seqoverlap 60) is a good trade-off between removing ambiguously aligned sites and keeping phylogenetically informative sites of the PKS sequences. The final data matrix consisted of 624 amino acid sequences with 1049 amino acid sites, 1049 distinct patterns, and 1043 parsimony-informative sites from 61 taxa (23 studied genomes and 38 other fungal and bacterial taxa from Kroken et al. [14]). In addition, PKS23 from *Stereocaulon alpinum* (QXF68953) and PKS16 from *Cladonia grayi* (ADM79459) from NCBI were included. The alignment is available on figshare (http://doi.org/10.6084/m9.figshare.19487837). 

We selected the best-fitting substitution model (LG+F+G4) according to the Akaike and Bayesian Information Criteria using the ModelFinder implemented in IQ-TREE [49] on our TI-PKS alignment. We calculated a phylogenetic tree using maximum-likelihood (ML) analysis implemented in IQ-TREE v2.1.4 [48], with 1000 ultrafast bootstrap replicates [59]. We also calculated a tree using RAxML-NG v1.0.3 [60] with parameters --all --bs-trees 100 --model LG+F+G4 --threads 16 --data-type AA. The inferred phylogenetic tree was then rooted with Bacterial Type II polyketide sequences using phyx [61].

To compare the congruence of the trees, we utilized Dendroscope v3.7.6 [62] using Tanglegram (Algorithms). The resulting tree was visualized using FigTree 1.3.1 [55] and a custom R script, with additional annotations added in Adobe Illustrator v24.0.3.

## 3. Results and Discussion

### 3.1. General Characteristics of De Novo Bacidia rubella Genome

We report the de novo assembled genome of the lichen-forming fungus *Bacidia rubella* obtained from an axenic fungal culture. Our hybrid approach to sequencing, using Illumina short-read with Oxford Nanopore long-read, resulted in a high-quality genome assembly of *B. rubella*. The final assembly had a size of 33.52 Mb in 51 scaffolds, an N50 of 1.77 Mb (Table 1), and was 98% BUSCO complete (fungi_odb9). Using multiple ab-initio gene-calling methods, 8773 genes were identified (Table 1). The similarity of standard genome metrics between *B. rubella* and *B. gigantensis* [63] suggests a high-quality *B. rubella* genome (Table 1).

The genome of *B. rubella* contains 31 BGCs (Table 2), including six non-reducing and four reducing TI-PKS sequences. This TI-PKS biosynthetic arsenal is similar to that of *B. gigantensis,* containing 11 identified TI-PKS genes (seven NR-PKSs and four R-PKSs, respectively; Table 2). Despite the overall similarity of TI-PKS gene numbers in *B. rubella* and *B. gigantensis*, our BLAST-based comparison of these biosynthetic clusters showed only two significant hits between the two species: one for R-PKS (bacgigpred_003963 and bacrubpred_000636), and one for NR-PKS (bacgigpred_008278 and bacrubpred_000202), with 62.8 and 63% similarity in amino-acid sequences, respectively. This result is not surprising, given that both fungal species differ in their secondary metabolite profiles. The only secondary compound of *B. rubella* is atranorin, which is the most widespread secondary metabolite found in the genus *Bacidia* s. lat. (Ekman 1996) [64]. In contrast, *B. gigantensis* is currently the only known *Bacidia* species to produce homosekikaic acid [65]. 

The two species *Ramalina intermedia* and *R. peruviana* belong to the same family as the genus *Bacidia* but differ in having many more BGCs compared to *B. gigantensis* and *B. rubella*. In detail, *R. intermedia* contains 54 BGCs, including thirteen non-reducing, seventeen reducing and one partially reducing TI-PKS sequences. *Ramalina peruviana*, on the other hand, contains 43 BGCs including nine non-reducing and seven reducing genes, and one partially reducing gene (Table 2).

#### 3.1.1. Atranorin

**Figure 2:**
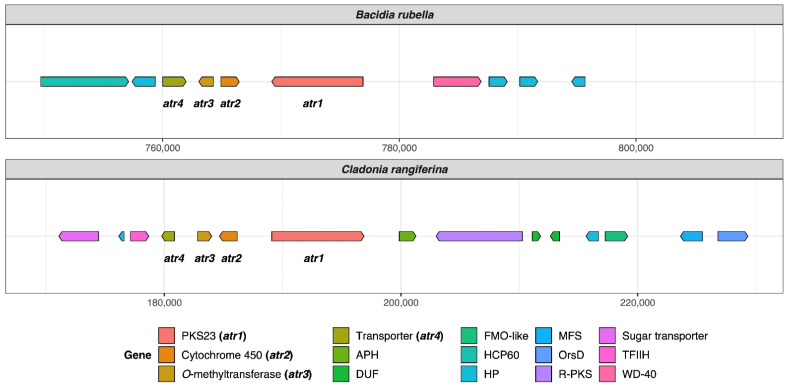
Organization of putative atranorin BGCs in *Bacidia rubella* and *Cladonia rangiferina*. The genes PKS23 (*atr1*), Cytochrome 450 (*atr2*), *O*-methyltransferase (*atr3*), and transporter gene (*atr4*) are present on the left side of the gene arrow plot. The BLAST percent similarity between two genes is 73% for *atr1*, 74% for *atr2*, 78% for *atr3*, and 91.5% for *atr4*. APH, phosphotransferase enzyme family (PF01636); DUF, domain of unknown function; FMO-like, flavin-containing monooxygenase family (PF00743); HCP60, HSP60 chaperone family (PF00118); HP, hypothetical protein; MFS, Major Facilitator Superfamily, transporter gene (PF07690); R-PKS, reducing PKS (in this case from R-VIII group); OrsD, Orsellinic acid/F9775 biosynthesis cluster protein D family (PF12013); TFIIH, Ssl1-like protein, subunit of the transcription factor II H complex (PF04056); Sugar_tr, Sugar transporter family (PF00083); WD-40, WD40 repeat (PF00400).

Atranorin is the main secondary compound known in *B. rubella* [64], and its biosynthetic pathway has received attention in previous studies on lichen-forming fungi [26,29]. Our phylogenetic results—which included previously identified sequences of genes involved in atranorin production from *Cladonia* species and *Stereocaulon alpinum*—revealed a putative PKS23 homolog in *B. rubella*. This is consistent with the phylogenetic investigation of Kim et al. [29], where PKS23 sequences were reported to group together with sequences from atranorin-producing lichen-forming fungi (see Section 3.3 Type I PKS phylogeny). The domain configuration of *B. rubella* PKS23 also supports our phylogenetic results, showing the same organization of putative atranorin BGC in *B. rubella* as has been reported for *Cladonia rangiferina* (Figure 2). The *B. rubella* PKS23 cluster contains a cytochrome P450 domain (*atr2*) required for oxidation, as well as an *O*-methyltransferase (OMT) domain (*atr3*) and transporter gene (*atr4*), which are involved in atranorin biosynthesis [29]. A BLASTp search of the OMT domain (*atr3*) in *B. rubella* PKS23 resulted in 36% protein sequence identity to Trt5 (UniProtKB accession no. Q0C8A3). This level of similarity is consistent with previous findings by Kim et al. [29] for *C. rangiferina* PKS23.

**Figure 3:**
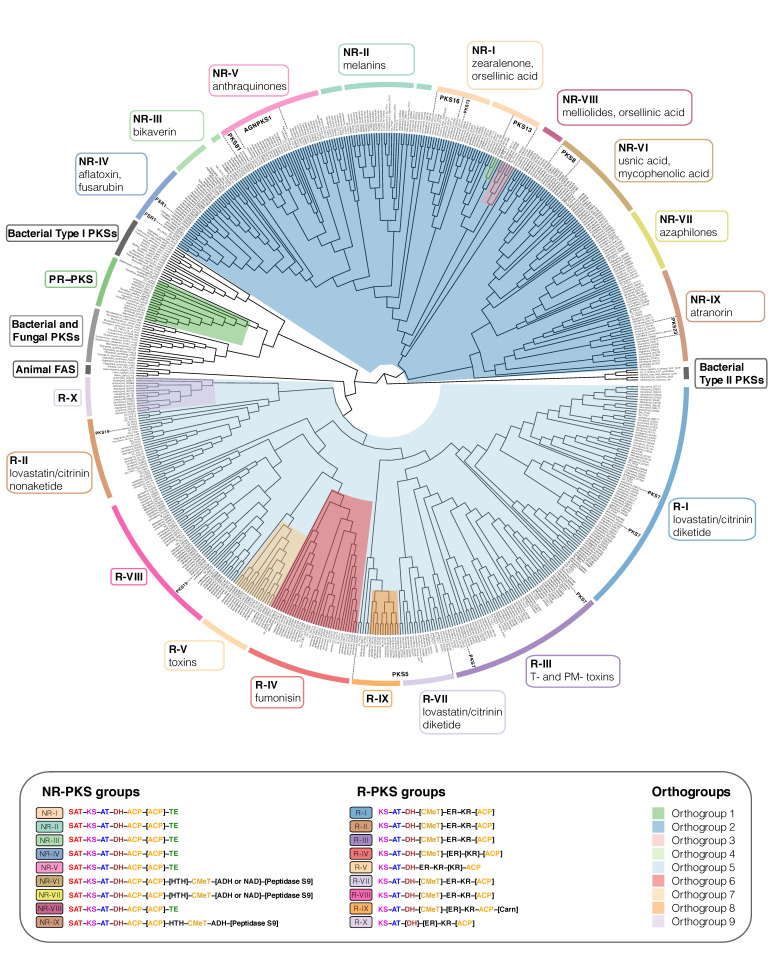
Maximum-likelihood phylogeny of Type I PKS genes inferred by IQ-TREE using Type II Bacterial PKSs as outgroup. Clades containing lichen-forming fungi are highlighted and the corresponding Orthogroups (1 to 9, respectively) are indicated by different colors. PR-PKS corresponds to Orthogroup 1 (green); the nine NR-PKS groups (NR-I to NR-IX) belong to Orthogroups 2, 3 and 4 (dark blue, pink and light green, respectively); the ten R-PKS groups (R-I to R-X) belong to Orthogroups 5, 6, 7, 8, and 9 (light blue, red, peach, orange, and lilac, respectively). Characteristic secondary substances for the groupings are given in the corresponding colored boxes. Groups not containing lichen-forming fungal genes are indicated by grey boxes. For each group, the domain arrangement of PKS is highlighted with distinct colors: SAT—starter unit-ACP transacylase; KS—ketoacyl synthase; AT—acyltransferase; ACP—acyl carrier protein; KR—ketoreductase; DH—dehydratase; ER—enoyl reductase; CMeT—methyltransferase; TE—thioesterase; HTH—helix-to-helix; ADH—adhydrolase; NAD—NAD-binding; Carn—Choline/Carnitine O-acyltransferase domain.

The newly identified, putative PKS23 sequence of *B. rubella* was recovered as a sister to PKS23 sequences from *C. rangiferina* and *S. alpinum,* suggesting a sister-group relationship of *Bacidia* with Cladoniaceae (See Figure 3 in Section 3.3: Type I PKS Phylogeny), whereby this clade formed a sister-group to the PKS23 sequences from Parmeliaceae (schematically shown in Figure 1).

This is in contrast to the family-level taxonomy previously established, e.g., by Peršoh et al. [66] and Miadlikowska et al. [50], where Cladoniaceae is more closely related to Parmeliaceae than to Ramalinaceae. To test the monophyly of the clade comprising *Bacidia* + Cladoniaceae compared to a group comprised of Parmeliaceae and Cladoniaceae, we performed a phylogenetic tree topology test on the PKS23 dataset. Specifically, we compared the unconstrained ML tree recovering *Bacidia* + Cladoniaceae as monophyletic (Topology 1) versus the constrained tree recovering Cladoniaceae + Parmeliaceae as monophyletic and *Bacidia* sister to that branch (Topology 2; Figure 1).

Our results showed that the topology constraining Cladoniaceae + Parmeliaceae as monophyletic is significantly less likely (0.8%, Figure 1, Table 3). Possible explanations are that the ancestor of *Bacidia* may have acquired the PKS23 gene from an ancestor of Cladoniaceae via horizontal gene transfer or that the PKS23 genes were generated by convergent evolution. It is obvious that these hypotheses require additional testing with augmented sampling of PKS23 sequences from Cladoniaceae, Parmeliaceae, and Ramalinaceae. Both scenarios may also explain the scattered occurrence of atranorin in Ramalinaceae.

Even though a PKS16 gene from *Cladonia grayi* was first shown to be involved in grayanic acid production by Armaleo et al. [18], a homolog of this gene from *C. rangiferina* was later suggested to be involved in atranorin production by Elshobary et al. [26]. In our phylogeny, these genes group together with genes from several other *Cladonia* and lichen-forming fungi (PKS16 clade, See Figure 3 in Section 3.3: Type I PKS Phylogeny), but not all of them are known as atranorin producers (Table 2). Moreover, atranorin is a B-orcinol depside, and a corresponding biosynthetic gene would require a CMeT domain to add a methyl group to the depside ring. This is not the case for PKS16, which is thought to be involved in the synthesis of orcinol depsides [25]. Our comparison of known metabolites in lichen-forming fungi containing PKS16 does not reveal a clear pattern either (See Figure 3 in section: Type I PKS Phylogeny, Group NR-I, PKS16 clade; Table 2). As such, the biosynthetic role of PKS16 genes remains elusive.

#### 3.1.2. Homosekikaic Acid

Homosekikaic acid is a major secondary metabolite in *Bacidia gigantensis* as well as in both *Ramalina intermedia* and *R. peruviana*. However, it has not been found in *B. rubella*. To our knowledge, there was no putative PKS reported as being involved in the biosynthesis of homosekikaic acid, and its biosynthesis has not been characterized using gene expression or heterologous expression experiments. To identify candidate genes involved in the biosynthesis of homosekikaic acid in these three species, we used BLASTp on all predicted BGC sequences from the two *Bacidia* and *Ramalina* species. We identified three BGC candidates with BLAST similarity ranging from 53 to 93%, but none of them were a TI-PKS. Instead, we recovered Type 3-PKS homologs from *B. gigantensis* (BGC 1.2), *R. intermedia* (BGC 6.1) and *R. peruviana* (BGC 223.1) as potential homosekikaic biosynthetic genes; however, none of *B. rubella* BGCs showed a high similarity to them. Furthermore, our TI-PKS phylogeny did not reveal any clade with both *Ramalina* species and *B. gigantensis* that excluded *B. rubella*. A possible explanation for the observed pattern is that the BGC responsible for synthesizing homosekikaic acid is present in the genome of *B. rubella,* but not expressed, and therefore homosekikaic acid is not produced in detectable amount. This hypothesis requires testing with gene expression experiments and analyses of substance profiles. 

#### 3.1.3. Other Biosynthetic Genes Identified In Silico in the Two *Bacidia* Species

Using further in silico analyses with antiSMASH, we were able to identify genes encoding enzymes to synthesize secondary metabolites such as clavaric acid (100% similarity) and squalestatin S1 (40% similarity). These terpenes have been identified in both *Bacidia*. Moreover, we identified a monascorubrin biosynthetic gene in *B. rubella* confirmed by 100% BLAST identity to the monascorubrin biosynthetic gene from *Talaromyces* (*Penicillium*) *marneffei* (PKS3: HM070047) [67]. Apart from monascorubrin biosynthesis, PKS3 genes were suggested to be involved in the production of the well-known toxin citrinin, as well as a yellow pigment, ankaflavin [67]. Monascorubrin and its related compounds are polyketides used as natural red colorants for food [68]. In *B. rubella*, monascorubrin could be responsible for the characteristic orange to the orange-brown coloration of the apothecia. However, these substances have not yet been reported from *B. rubella*.

In the genome of *B. gigantensis*, we identified genes possessing high sequence similarity with genes involved in the production of pyranonigrin E (100% similarity to BGC0001124), naphthopyrone (100% similarity to BGC0000107), and melanin (100% similarity to BGC0001265). However, in most cases, only a part of the sequences showed a high percentage of similarity; therefore, our in-silico based report is provisional. Additional detailed studies are necessary to confirm these first functional assignments.

### 3.2. Biosynthetic Gene Composition in Twenty-Three Annotated Fungal Genomes 

The basic statistics for all twenty-three studied genomes are provided in Table 4. According to BUSCO homology searches against the fungal dataset (fungi_odb9), most genomes were highly gene complete. We included only two genomes with completeness below 90%, namely *Alectoria sarmentosa* (75.8%) and *Graphis scripta* (88.6%). The number of predicted genes was in the range of 6756 to 11,072. The lowest number of predicted genes was observed for *Ramalina peruviana* (most likely due to issues in the quality of the assembly given the lower sequencing depth of 2×), and the highest number was observed for *Evernia prunastri* (Table 4).

We investigated the BGCs predicted in all twenty-three studied genomes that belong to different taxonomic groups and synthesize a plethora of secondary metabolites (Table 2; Table 4). Our results revealed a high number of BGCs, with an average of 48 clusters per genome. The smallest number was recovered in the genome of *Lasallia pustulata* (26 BGCs), and the highest in *Evernia prunastri* (86 BGCs), which agrees with the results reported by Calchera et al. based on 15 lichenized genomes [6]. In nearly half the genomes, NR-PKSs are more common than R-PKS (Table 2). This evidence is in contrast to previous results reporting that R-PKS gene numbers exceed the number of NR-PKS genes [6]. As such, a larger data set is necessary to confirm whether R-PKS or NR-PKSs are more numerous in the genomes of lichen-forming fungi. The total number of TI-PKSs identified across all studied genomes was 478. Of those, 44.35% were of the non-reducing (NR), 53.35% of reducing (R), and 2.3% of partly reducing (PR) PKSs. The highest number of PKS genes was found in *E. prunastri* (36 TI-PKS clusters) and *C. rangiferina* (34 clusters) and the lowest numbers were in *B. rubella* (10 TI-PKS clusters), *B. gigantensis* (11 clusters), and *Cyanodermella asteris* (10 clusters) (Table 2).

Our broad genomic sampling provides phylogenetic context for the NR-PKS genes of *B. rubella* and *B. gigantensis*. The six TI-PKS genes from *B. rubella* are NR-PKSs (three in Subclade I and three in Subclade II), while for *B. gigantensis* seven TI-PKS genes are NR-PKSs (all are in Subclade I).

The discrepancy between the large number of recovered TI-PKS sequences and the few experimentally verified secondary metabolites (Table 2) raises questions about the role these genes play in the secondary metabolism, and about the products they produce (e.g., [9,24,25,29]. However, results linking PKS genes to lichen secondary metabolites beyond in silico methods are still scarce.

### 3.3. Type I PKS Phylogeny

Our maximum-likelihood phylogeny of TI-PKS with a total of 624 sequences recovered from twenty-three fungal genomes and supplemented with previously published sequences is the largest analysis of biosynthetic gene content of various TI-PKS genes in lichen-forming fungi to date. 

The TI-PKSs phylogenetic tree is divided into six main subgroups (Figure 3): Bacterial Type II PKS (including bacterial and mitochondrial ketoacyl-ACP-synthetases; used as outgroup), Bacterial Type I PKS (also including some fungal sequences), animal fatty acid synthase (FAS), PR-PKS, NR-PKS, and R-PKS. Lichen PKS genes are distributed across the three of these main subgroups, viz. PR-PKS, NR-PKS, and R-PKS. These groups were also recognized by Kroken et al. [14] and Calchera et al. [6], but both used the single KS domain for tree reconstruction, and the latter subsumed PR-PKS and R-PKS.

- *TI-PKS domain content mostly corresponds to phylogeny*

TI-PKS genes encode for multi-domain enzymes, with each domain executing a specific function. The order and domain content of the PKSs thus defines the class of polyketides produced by the corresponding BGC. The presence or absence of individual domains has led to the classification of fungal PKSs into three main subgroups. The first subgroup comprises NR-PKSs, which lack reductive domains. The generalized domain content of NR-PKS in fungi studied here is SAT-KS-AT-DH-ACP-[ACP]-HTH-CMeT-[TE or ADH or NAD]-[Peptidase S9] (NR-I to NR-IX; Figure 3). 

The second subgroup comprises PR-PKSs containing a single KR domain or KR and DH domains. The generalized domain content in PR-PKS according to our phylogeny is KS-AT-[DH]-KR-ACP (PR-PKS; Figure 3). 

Finally, the third subgroup—R-PKSs—contains a complete set of reductive domains, viz. KR, DH, and ER and thus exhibit the following domain structure: KS-AT-DH-CMeT-ER-KR-ACP-[Carn] (R-I to R-X; Figure 3). Additional domains, such as helix-to-helix (HTH), adhydrolase (ADH), NAD-binding (NAD), Peptidase S9, and Choline/Carnitine O-acyltransferase domain (Carn) were not shown in the previous studies of TI-PKS in lichen-forming fungi. We discuss them in detail below in the corresponding sections.

- *The discrepancy of grouping PKS genes*

Assigning PKS genes to different groups in fungi on the account of gene domain composition and their synthesized products was introduced by Kroken et al. [14] and later refined based on DH-domain pocket sizes by Ahuja et al. [69] and Liu et al. [70]. This classification was used in several studies investigating PKS gene diversity in lichen-forming fungi, e.g., [29,71]. Despite this existing classification, it remains unclear if all groups indeed form monophyletic clades and if all genes from one group synthesize the same (or at least chemically similar) substances. In our phylogeny, previously proposed groups were not always monophyletic (e.g., NR-II; Figure 3), and experimentally characterized genes from one group have been shown to produce different substances (e.g., PKS81 and AGNPKS1 in NR-V; Figure 3). Additionally, we performed an ortholog clustering using Orthofinder on all TI-PKS sequences to provide an objective way of identifying groups of sequences. This resulted in nine orthogroups (Figure 3: colored clades), named Orthogroup 1 to Orthogroup 9, respectively. Orthogroup 1 corresponds to the PR-PKSs (Figure 3). NR-PKSs are spread across Orthogroups 2, 3, and 4 but none of them correspond explicitly to any of the nine groups identified before by Pizarro et al. [71] and Kim et al. [29]. Orthogroups 5, 6, 7, 8, and 9 contain R-PKSs, where only Orthogroup 6 (R-IV) and 7 (R-V) agree with groups defined in Kroken et al. [14] and Punya et al. [72]. However, all Orthogroups identified here form well supported clades in the PKS phylogeny (Figure 3). The discrepancy between our results and previous studies needs to be investigated in subsequent studies. In our phylogeny, several groups of the NR-PKS and R-PKS showed differences in the PKS domains present corresponding to the supported clades; these groups do contain support from different sources and thus merit discussion (see below).

#### 3.3.1. Lichen-Forming Fungi Contain Only a Few Partially Reducing PKSs in the Phylogenetic Neighbourhood to Bacterial PKSs

The PR-PKS sequences formed a well-supported clade sister to bacterial Type I PKS sequences with other fungal PKSs (PR-PKS; Figure 3). Their domain configuration is KS-AT-[DH]-KR-ACP, including only a single reductase domain (KR). In contrast to the large NR- and R-PKS groups, PR-PKS contains genes mainly from *Aspergillus* and *Penicillium,* and only a few genes from lichen-forming fungi. Genes from the studied fungal genomes have the typical PR-PKS domain composition, but in two genes from two *Ramalina* (ramintpred_001715 and ramperpred_002012), the DH domain was missing. PR-PKS genes form a sister clade to Bacterial Type I PKSs (Figure 2). Bacterial T1-PKSs often possess a KR domain catalyzing the first step in the reductive modification of beta-carbonyl centers in the growing polyketide chain. This domain requires NADPH to reduce the keto- to a hydroxy group [73]. 

The “simple” domain configuration of Bacterial and PR-PKS genes compared to NR- and R-PKSs has not escaped our attention. Our study was not designed to specifically test how PKS genes in Ascomycetes were acquired and how they diversified. However, the placement of Bacterial and NR-PKS sequences as the earliest branches in our phylogeny supports the hypothesis suggested by Kroken et al. [14] that fungal TI-PKS genes could have been acquired by an ancient horizontal gene transfer event between bacteria and fungi. Additionally, our results suggest that ancestral TI-PKS genes may have been partially reducing. Under this scenario, NR- and R-PKS evolution could have been connected to domain structure modification (such as gaining SAT in NR-PKS) and subsequent functional diversification. However, this cannot be concluded with certainty without a greatly expanded sample of genomes from other fungal groups, including genomes from early branching fungal lineages and comprehensive ancestral state reconstructions.

#### 3.3.2. Fungal PKSs Producing Non-Reduced Polyketides

##### The Diversity of NR-PKS Sequences

In previous studies, NR-PKSs were divided into nine major groups based on protein sequence similarity and PKS domain content [14,29,69,71]. Similar to previous studies, we observed characteristic domain configurations for the different clades in our phylogeny. The domain structure of NR-PKSs in the studied fungi can be generalized as SAT-KS-AT-DH-ACP-[ACP]-HTH-CMeT-[TE or ADH or NAD]-[Peptidase S9]; however, some PKS genes may deviate from this structure. The observed domain content variations are not random but rather occur in two main patterns: either through the duplication of the ACP domain, or through the addition of an N-terminal TE domain. PKS genes possessing an additional ACP domain are scattered throughout different NR-PKS clades, suggesting multiple independent gains. However, the functional significance of ACP domain duplications is still unknown [14]. 

Most of the NR-PKS sequences belong to a large clade containing groups NR-I to NR-V and NR-VIII, with domain configuration containing a TE domain at the N-terminal end.

The irregularly present additional TE domain is involved in a thioesterase-mediated product release, which is the most common release mechanism in TI-PKS [74]. It regularly extends to a C-C Claisen cyclization domain (TE/CLC domain), e.g., in *Aspergillus parasiticus* PksA [75]. Although CYC domains have previously been reported from various fungi [14,69,70], we could not identify them in our analyses and thus did not indicate them in the phylogeny.

##### Subclade I of NR-PKSs (Including Groups NR-I to NR-V, and NR-VIII)

Group NR-I

The paraphyletic group NR-I contains several clades with previously characterized sequences involved in the biosynthesis of aromatic compounds derived from orsellinic acid, such as grayanic acid (PKS16), physodic acid and olivetoric acid [24,25], lecanoric acid [76], xanthones, aflatoxin, and naphthoquinones [14]. In addition, the clade also comprises PKS13 sequences from several lichen-forming fungi and *Gibberella zeae* as well as a PKS15 sequence from *Botryotinia fuckeliana* with unknown functions.

Group NR-II

Our results show that NR-II is a diverse group, comprised of several clades containing sequences from lichen-forming fungi interspersed with characterized PKS genes proposed to be involved in the biosynthesis of various substances. The earliest branching clade contains genes involved in the 6-hydroxymellein biosynthesis (NR-II; Figure 3), a key intermediate in terrein biosynthesis [77]. Terrein, produced in large quantities by *Aspergillus terreus*, has phytotoxic activity and has the potential to serve as a novel antibiotic [78]. Our phylogeny revealed sequences of four lichen-forming fungi (clagrapred_001463, clauncpred_002890, psefurpred_002946, and grascrpred_004317) clustering together with *terA* gene in *A. terreus*. A BLAST search of *terA* (GenBank: EAU38791) against these sequences revealed 58 to 67% similarity at the amino acid level, indicating a high degree of conservation despite an approximate 350-million-year split of Eurotiomycetes (*Aspergillus*) and Lecanoromycetes [79]. Terrein has not been reported from lichen-forming fungi; therefore, without further experiments and detailed substance characterization, terrein production in lichen-forming fungi remains hypothetical.

Another important pattern is the occurrence of characterized melanin genes and melanin precursors in different clades. Melanins are a diverse group of substances that play a role in virulence, morphogenesis or the response to environmental stress, and can be synthesized via different pathways [80]. PKSs produce two forms of melanin: either 1,8-dihydroxynaphtalene (DHN) melanin or Deoxybostrycoidein-melanin [81]. Melanins are insoluble and thus cannot be studied by standard biochemical methods [80]. In the PKS gene survey by Kroken et al. [14], all melanin synthesizing genes were grouped together in a single monophyletic clade. In our analysis, they are recovered in at least three clades containing sequences from several Lecanoromycetes and *Endocarpon pusillum*. Sister to NR-II is a clade containing sequences from *Colletotrichum lagenarium* (PKS1), *Glarea* sp., *Nodulisporium* sp., and *C. heterostrophus* (PKS18), which were also characterized as producers of melanin [14]. The high number of genes from lichen-forming fungi close to characterized melanin biosynthetic genes suggests an essential role of melanins in lichens.

Groups NR-III and NR-IV

The sister groups NR-III and NR-IV contain sequences of proteins producing large polyketide chains, such as conidial yellow pigment Alb1 (NR-III) or aflatoxin/sterigmatocystin of *A. nidulans* (NR-IV) [14,71,82]. 

NR-III additionally contains characterized PKSs producing duclauxin (35–71% similarity) and naphthopyrone (100% similarity). Duclauxins are dimeric, heptacyclic fungal polyketides with various activities [83], while bis-naphthopyrones act in herbivore defense in filamentous ascomycetes [84].

Several genes recovered in the NR-IV group are potentially involved in producing different fungal pigments. The genes from *Ramalina intermedia* (ramintpred_005964) and *Cladonia grayi* (clagrapred_006320) were assigned to an FSR1 gene, which is involved in producing highly pigmented naphthoquinones fusarubins responsible for fruiting body coloration of *Fusarium fujikuroi* [85]. A BLAST search of the *R. intermedia* gene (ramintpred_005964) revealed 72.3% similarity to a PKS of *Cladonia metacorallifera* (GenBank: QIX11499) that is involved in the biosynthesis of the red compound cristazarin, characterized by antibacterial and antitumor activity [86]. Sequences of *Bacidia rubella* and several *Cladonia* species producing red pigments were recovered sister to the putative *Ramalina intermedia FSR1* gene. Interestingly, *R. intermedia*, at the same time, lacks red pigments [87]. This close phylogenetic proximity raises the possibility that combinations of substances synthesized by different PKS genes could be responsible for red-colored pigments (see also discussion on monascorubrin above), particularly for the red fruiting bodies in *B. rubella*.

Group NR-V

According to the previous studies, NR-V contains PKSs without the TE domain [71]; however, in the characterized PKSs in our phylogeny, the TE or R domains were present in all clades. The genes in the NR-V group are suggested to be involved in the production of different mycotoxins, such as desertorin (*Aspergillus nidulans*) and atrochrysone (*Aspergillus fumigatus*) [71,88,89].

The crown clades in the NR-V group include the annotated PKS81 orthologs from *Cladonia metacorallifera*, *C. macilenta*, *Lasallia pustulata* and *L. hispanica* as sister to a PKS19 sequence from *Cochliobolus heterostrophus* and two clades with characterized Agnpks1 also from several lichen-forming fungi. Agnpks1 (Atrochrysone carboxylic acid synthase) was originally described from *Penicillium divaricatum* and is involved in the biosynthesis of agnestins and dihydroxy-xanthone metabolites [90]. Xanthones and related benzophenones are produced by various filamentous fungi. They exhibit insecticide, antioxidant, antibacterial, anti-inflammatory, and anticancer activities [91]. Examples include desmethyl-sterigmatocystin, a key intermediate of the aflatoxin group of mycotoxins produced by *Aspergillus flavus*, and a norlichexanthone from the lichen-forming *Lecanora straminea* [90]. An additional BLAST search of norlichexanthone synthase from *L. straminea* (GenBank: D7PI15) against our annotated fungal sequences showed at least 37% similarity to sequences from the clade containing PKS81 and Agnpks1. The relationship between these two genes remains unclear, but given our results, PKS81 might belong to or have evolved from Agnpks1. Additional closer investigations will be necessary to show the role of PKS81 and Agnpks1 orthologs in lichen-forming fungi and their possible role in xanthone biosynthesis.

##### Subclade II of NR-PKSs (Including Groups NR-VI, NR-VII, and NR-IX)

The second largest clade of NR-PKSs includes the previously recovered groups NR-VI, NR-VII, and the recently defined group NR-IX [29] (Figure 3). The generalized domain arrangement in these groups is SAT-KS-AT-DH-ACP-[ACP]-[HTH]-CMeT-[ADH/NAD]-[Peptidase S9], deviating from the generalized arrangement for other NR-PKS (see above). Although previously reported from all NR-PKS groups except group NR-V [69,71], we did not observe TE domains in groups NR-VI, NR-VII, and NR-IX. Instead, we observed NAD and ADH domains—in some cases followed by a Peptidase S9 (PFAM: PF00326) domain. Peptidase S9 belongs to proteolytic enzymes that consume serine in their catalytic activity, and they are ubiquitously found in viruses, bacteria, and eukaryotes [92]. We also observed a helix-turn-helix domain (HTH) being inserted after one or two ACP domains in several groups. The HTH domain was previously found in PKSs in fungi but it has not been shown in the arrangement of the NR-PKSs of lichen-forming fungi before. Proteins with an HTH motif are, for example, involved in DNA repair, RNA metabolism, and protein–protein interaction [93], and could thus be involved in developmental or morphogenic processes.

Group NR-VI

Characterized PKSs in this group include PKS18 and PKS19 genes from *Botryotinia fuckeliana.* Genes from this group were suggested to be involved in usnic acid biosynthesis [71], and according to Kim et al. [29] they belong to PKS8. Our results show that this group is divided into two clades. The first clade contains genes of known usnic acid producers such as *Ramalina intermedia*, *R. peruviana*, *Cladonia metacorallifera*, *C. macilenta*, *C. uncialis*, *Evernia prunastri*, *Alectoria sarmentosa*, *Letharia columbiana,* and *Usnea hakonensis*, interspersed with genes from lichen-forming fungi known not to produce usnic acid. The second clade includes genes mostly from lichen-forming fungi not producing usnic acid, including a gene from *B. rubella* (bacrubpred_000551). However, sequences from *Cladonia macilenta*, *C. uncialis,* and *Evernia prunastri* were also recovered in this clade.

Group NR-VII

The only genes characterized in this group are PKS17 from *Botryotinia fuckeliana* and PKS21 from *Cochliobolus heterostrophus*. These genes have been proposed to produce citrinin or lovastatin [14,94,95]. The other identified genes from the lichen-forming fungi in this group had 50 to 70% BLAST similarity to genes involved in the biosynthesis of monascorubrin, citrinin, conidial yellow, azanigerone A, and stipitatic acid (see also discussion on monascorubrin above). As mentioned above, the biosynthetic gene from *B. rubella* (bacrubpred_007642) has a 100% identity to the monascorubrin biosynthetic gene.

Group NR-IX

The recently recognized group NR-IX [29] contains several predicted PKS23 sequences, involved in the biosynthesis of atranorin (see sections on 3.1.1 Atranorin and 3.1.2 Homosekikaic acid above) and methylated orsellinic acid derivatives. In contrast to the groups NR-VI and NR-VII, NR-IX sequences always contain an ADH and lack a NAD domain after CMeT, while Peptidase S9 is only sporadically present. It is necessary to note that only one ACP domain was present in the PKS23, including atranorin producers. In addition, other characterized PKSs of this group are from *Cochliobolus heterostrophus* (PKS22 and PKS23) and *Botryotinia fuckeliana* (PKS16 and PKS20). 

#### 3.3.3. Fungal PKSs Producing Reduced Polyketides

##### The Diversity of R-PKS Sequences

R-PKS genes are involved in synthesizing various reduced, usually linear polyketides. Many are precursors of toxins that are active in animals (e.g., lovastatin, citrinin, and fumonisin) or plants (e.g., T-toxin and PM-toxin) [96,97,98,99]. We follow this classification in our discussion whenever possible, while also highlighting deviations. Compared to NR-PKSs, less is known about the possible roles of R-PKSs. In our phylogeny, R-PKSs formed a large clade sister to NR-PKSs with many well-supported subclades. Unlike what has been shown in previous studies, all studied R-PKSs here lacked a second ACP domain, and in some cases, ACP was missing. According to Kroken et al. [14], four major groups (R-I to R-IV) of R-PKS genes can be distinguished based on their overall domain structure and produced compounds. This classification was later extended to groups R-V to R-VIII by Punya et al. [72]. Over 70% of the studied fungal sequences could be assigned to groups designated by Kroken et al. [14] and Punya et al. [72] with the general domain content: KS-AT-DH-[CMeT]-ER-KR-[ACP]. Two clades recovered by Punya et al. [72] are missing from our tree, namely R-VI and R-VIII. Clade VI contained uncommon PKS-NRPS hybrids with four additional domains after ACP, viz. condensation (C), adenylation (A), thiolation (T), and reductase (R) [72]. As our study focused on the TI-PKSs, it did not include hybrid NRPS-PKS genes. The sequences from R-VIII were not present in our phylogeny, and are thus not feasible to identify with confidence. 

Based on our phylogenetic results, we identified two R-PKS clades that did not match any of the R-PKS groups reported in previous studies. Sequences in those two clades had characteristic domain structures and were recovered as distinct orthogroups in our Orthofinder analysis. This leads us to propose these clades as new reducing PKS groups, R-IX and R-X, following the numbering scheme by Kroken et al. [14] and Punya et al. [72]. 

##### Groups of R-PKSs

Group R-I

Group R-I is the largest group of R-PKSs in our tree. It contains one of the R-PKS genes synthesizing the diketide portion of lovastatin and citrinin, T-toxin, and PM-toxin [14]. Our results show that the genes of this group, recovered in a monophyletic clade by Kroken et al. [14], were nested in different clades in our analysis; therefore, R-I assignment is not complete.

Group R-II

Group R-II forms a well-supported clade and it was previously characterized by the absence of an ER domain [14]. In our phylogeny, however, ER domain was present in all lichen-forming fungi. The group includes many previously published sequences and orthologs from *Cladonia* and *Letharia* species, *Xanthoria elegans*, *Pseudevernia furfuracea,* and *Ramalina intermedia*. The *R. intermedia* ortholog was assigned to PKS19. It was predicted to be involved in the production of heptaketides with a role in a lesion of rice leaves formation in *Magnaporthe oryzae* [100]. Two characterized proteins from *A. terreus* lovB and *P. citrinum* mlcA synthesize the cyclic nonaketide portion of lovastatin and citrinin.

Group R-III

Group R-III forms a clade comprised of four sequences previously assigned to this group by Kroken et al. [14], and a sequence from *Cyanodermella asteris*. PKSs from this clade do not possess a CMeT domain. The experimentally characterized gene in group R-III is *C. heterostrophus* PKS2, which, along with PKS1, is required for the synthesis of T-toxin [14]. This group contains several sequences from Kroken et al. [14] interspersed with sequences from various lichen-forming fungi.

Group R-IV

Genes belonging to R-IV may contain a conserved CMeT domain. Thus, their domain configuration somewhat resembles that of groups R-I and R-II. However, N-terminal domains ER, KR, and ACP can be present or absent in R-IV. The only characterized PKS in this group is *G. moniliformis* FUM1 (Gm_FUM1_AAD43562), which makes the linear polyketide precursor of the toxin fumonisin [98]. This group contains several sequences from Kroken et al. [14] interspersed with sequences from various lichen-forming fungi.

Group R-V

Sequences in group R-V have similar domain composition like sequences from Orthogroup 7: KS-AT-DH-ER-KR-[KR]-ACP. However, a second KR domain is only present in *Dibaeis baeomyces* (dibbaepred_000831). We recovered genes from several lichen-forming fungi in this group. Again, experimentally characterized genes from *Gibberella moniliformis* (Gm_PKS13) and *Botryotinia fuckeliana* (Bf_PKS10) also recovered here indicate a possible role in toxin biosynthesis (Figure 3) [14].

Group R-VII

This group contains several previously characterized sequences as well as sequences from *R. intermedia*, *P. furfuracea*, *G. furfuracea,* and *C. macilenta* where we could assign PKS5 annotations. Additional sequences from lichen-forming fungi also recovered here had no functional annotations; as such the role of these genes remains unclear. The characterized sequences of *Aspergillus* (*Aspergillus_terreus*_*lovF*) and *Penicillium* (*Penicillium_citrinum_mlcB*) indicate a role in the production of the diketide portion of lovastatin and citrinin, but they were assigned to group R-I by Kroken et al. [14].

Novel group R-IX

A new group of reducing PKSs can be delineated based on a specific domain composition and our orthogroup assignment. We tentatively call this group R-IX following the naming scheme of Kroken et al. [14] and Punya et al. [72]. R-IX is sister to group R-VII and differs from all other groups by having a facultative Choline/Carnitine O-acyltransferase domain (Carn) following the ACP domain: KS-AT-DH-[CMeT]-ER-KR-[ACP]-[Carn] (Figure 3). The Carn domain can be found in several eukaryotic acetyltransferases and also at the C terminus of a highly reducing polyketide synthase (SdnO), where it is part of a gene cluster mediating the biosynthesis of glycoside antibiotics sordarin and hypoxysordarin [101].

Novel group R-X

Similar to R-IX, a second group of reducing PKSs can be identified based on a specific domain composition and orthogroup assignment. This group R-X, recovered as Orthogroup 9, contains only genes from lichen-forming fungi. Together with Group R-II, they share a sister-group relationship to all other R-PKSs in our phylogeny (Figure 3). Genes from this group may not have DH, ER and ACP domains, resulting in the following domain content: KS-AT-[DH]-[ER]-KR-[ACP]. Most fungal genes recovered here are missing the DH and ER domain. However, the genes from *Lasallia hispanica* (lashispred_001760) and *Evernia prunastri* (eveprupred_001763) contain a complete domain configuration: KS-AT-DH-ER-KR-ACP.

## 4. Conclusions and Limitations

This study presents a comprehensive analysis of the PKS gene content of the de novo sequenced genome of *B. rubella* and twenty-two publicly available genomes of mainly lichen-forming fungi. Our results reveal that a BGC including PKS23 is likely involved in biosynthesis of atranorin in *B. rubella*. Similar to previous studies (e.g., [6,23,24,29]), we observed a large diversity of PKS genes in lichen-forming fungi as well as *B. rubella*, much larger than expected based on their recorded secondary metabolite profiles. This highlights the potential of lichen-forming fungi to produce a much higher number of substances than previously assumed, as mentioned by Calchera et al. [6] focusing on foliose lichens. Our in silico approach provides first insights into the possible roles of PKS genes in crustose *B. rubella*; however, we are aware that this comes with limitations. Only detailed studies of individual BGCs combining gene expression analyses with analytical chemistry and metabolomics will allow thorough testing of the hypotheses proposed here. The prospects for this are promising as an increasing number of studies succeeded in the heterologous expression of lichen-forming fungal genes, (e.g., [29,76,86,102,103]). Subsequent in vivo studies of metabolite profiles in lichen-forming fungi supplemented by high-resolution MS/MS spectra [104] or metabolic profiling based on stable isotope analysis [105] will contribute to our knowledge on how secondary metabolites in lichen-forming fungi are produced and what roles they play. Combined with the growing number of available lichen-forming fungal genomes, we will be able to refine our understanding and formulate novel hypotheses about the biosynthetic potential of lichens.

## Figures and Tables

**Table 1:** Starting material, sequencing strategy and genome quality statistics of Ramalinaceae genomes analyzed in this study.

Nuclear Genome	*Bacidia rubella*	*Bacidia gigantensis*	*Ramalina intermedia*	*Ramalina peruviana*
**Substrate**	bark	bark	rock	twig
**Growth form**	crustose	crustose	fruticose	fruticose
**Source**	Axenic culture	Whole thallus	Whole thallus	Axenic culture
**Sequencing method**	Illumina NovaSeq SP; MinION	PromethION 24	Illumina MiSeq	Illumina MiSeq
**Raw reads produced**	17 Gbp; 22 Gbp (roughly)	32 Gbp	13.33 Gbp	-
**Coverage**	500×	500×	290×	2.0×
**Assembly size (Mb)**	33.52	33.11	26.19	25.53
**Largest scaffold (bp)**	2,353,056	3,530,911	898,913	694,821
**Average scaffold (bp)**	657,358	1,379,912	148,821	25,764
**Number of scaffolds**	51	24	176	991
**N50**	1,771,855	1,807,239	282,362	43,940
**GC (%)**	45.28	44.67	51.90	50.58
**Num of predicted genes**	8773	8451	7405	6756
**Num of predicted proteins**	8728	8400	7355	6706
**Number of unique proteins**	2514	2343	1099	1088
**tRNAs**	45	51	50	50
**Proteins with at least one ortholog**	5860	5711	6111	5467
**Single-copy orthologs**	2345	2345	2345	2345
**Secreted proteins (SignalP)**	678	612	464	384

**Table 2:** Overview of biosynthetic gene clusters and polyketide synthase families found in the twenty-three studied fungal genomes. The occurrence of the major secondary substance of *B. rubella*, atranorin, is highlighted. All sequences except the de novo sequenced *B. rubella* genome are from NCBI, if not otherwise specified in the first column.

Species (NCBI)	Species Tag	Genome Size (Mb)	No of Clusters	Type I PKS (Total)	Type I NR-PKS	Type I R-PKS	PR-PKS	Hybrid PKS-NRPS	NRPS/ Putative NRPS	Metabolites Reported
*Alectoria sarmentosa* (ASM973377v1)	alesarpred	39.5	36	12	5	7	0	1	2/12	Usnic acid, alectoronic acid (major), thamnolic, squamatic and barbatic acids
*Bacidia gigantensis* (ASM1945646v1)	bacgigpred	33.1	31	11	7	4	0	1	5/10	Homosekikaic acid
*Bacidia rubella* (this study)	bacrubpred	33.5	31	10	6	4	0	0	3/7	**Atranorin**
*Cladonia grayi* (JGI: Cgr/DA2myc/ss v2.0)	clagrapred	34.4	48	21	8	12	1	0	2/11	4-O-demethylgrayanic acid, colensoic acid, confumarprotocetraric acid, divaronic acid, fumarprotocetraric acid, grayanic acid, protocetraric acid, stenosporonic acid
*Cladonia macilenta* (Clmac_v1)	clamacpred	36.8	55	25	15	10	0	2	3/9	Thamnolic acid, barbatic acid, didymic acid, squamatic acid, usnic acid, rhodocladonic acid
*Cladonia* *metacorallifera* (KoLRI002260_v2)	clametpred	36.6	51	24	10	14	0	1	1/11	Usnic acid, didymic acid, squamatic acid, rhodocladonic acid
*Cladonia rangiferina* (ASM614605v1)	claranpred	34.5	68	34	14	20	0	2	3/12	**Atranorin**, protocetraric acid, fumarprotocetraric acid
*Cladonia uncialis* (ASM292778v1)	clauncpred	30.7	61	30	15	14	1	2	1/11	Usnic acid, squamatic acid
*Cyanodermella* *asteris* (Astra)	cyaastpred	28.6	35	11	5	5	1	1	3/10	Astin, skyrin
*Dibaeis baeomyces* (JGI)	dibbaepred	34.2	55	27	11	15	1	0	4/14	Baeomycic acid, squamatic acid
*Endocarpon* *pusillum* (Z07020)	endpuspred	36.2	32	14	4	9	1	1	3/4	Not reported
*Evernia prunastri* (ASM318436v1)	eveprupred	40.2	86	36	16	19	1	4	3/20	Usnic acid, **atranorin**, and chloroatranorin, evernic acid
*Graphis scripta* (JGI: CBS 132367)	grascrpred	34.8	54	21	6	15	0	1	6/14	Not reported
*Gyalolechia* *flavorubescens* (KoLRI002931)	gyaflapred	34.4	41	16	8	8	0	1	3/8	Parietin, emodin, fallacinal, fragilin
*Lasallia hispanica* (ASM325442v1)	lashispred	39.7	28	15	8	6	1	1	0/3	Gyrophoric acid, lecanoric acid, umbilicaric acid, skyrin
*Lasallia pustulata* (ASM863619v1)	laspuspred	32.9	26	17	9	7	1	0	0/4	Gyrophoric acid, lecanoric acid, hiascinic acid, skyrin
*Letharia columbiana* (Lecol_v1.0)	letcolpred	52.2	43	14	7	7	0	2	3/6	Vulpinic acid and **atranorin**
*Letharia lupina* (Lelup_v1.1)	letluppred	49.2	48	18	11	7	0	2	3/11	Vulpinic acid and **atranorin** in the cortex, with norstictic acid in the hymenium of the apothecia
*Pseudevernia* *furfuracea* (Pfur_oli_TBG_2151)	psefurpred	37.8	48	26	8	18	0	3	3/11	**Atranorin**, physodic acid and oxyphysodic acid
*Ramalina intermedia* (RamPxa02_v1.0)	ramintpred	26.2	54	31	13	17	1	3	5/9	Usnic acid (major); medulla with homosekikaic acid (major), sekikaic acid (major), 4’-O-methylnorhomosekikaic acid (minor)
*Ramalina peruviana* (RamPxa01_v1)	ramperpred	25.5	43	17	9	7	1	1	4/10	Usnic acid, homosekikaic acid (major), sekikaic acid (major), and 4’-O-methylnorhomosekikaic acid and 4’-O-methylnorsekikaic acid (minor)
*Usnea hakonensis* (Uhk_1.0)	usnhakpred	40.4	70	23	10	12	1	3	5/22	Usnic and norstictic acids
*Xanthoria elegans* (ASM1131630v1)	xanelepred	44.2	63	25	7	18	0	1	7/17	Parietin (major), fallacinal, emodin, teloschistin and parietinic acid

**Table 3:** Parameters for the topology test. Topology 1, recovering *Bacidia* + Cladoniaceae as monophyletic; Topology 2, recovering Cladoniaceae + Parmeliaceae as monophyletic. Value for the Kishino–Hasegawa test (Kishino and Hasegawa, 1989), Shimodaira and Hasegawa, 1989), expected likelihood weights (Strimmer and Rambaut, 2002) and approximately unbiased (AU) test (Shimodaira, 2002) are given.

Tree	logL	Deltal	p-KH	p-SH	c-ELW	p-AU
**Bacidia + Cladoniaceae**	−28,054.52811	2.062 × 10^−8^	0.495+	0.814+	0.496+	0.566+
**Cladoniaceae + Parmeliaceae**	−28,108.54142	54.013	0.0126−	0.0126−	0.00853−	0.0081−

deltaL: logL difference from the maximal logl in the set. p-KH: *p*-value of one-sided Kishino–Hasegawa test (1989). p-SH: *p*-value of Shimodaira–Hasegawa test (2000). c-ELW: Expected Likelihood Weight (Strimmer and Rambaut 2002). p-AU: *p*-value of approximately unbiased (AU) test (Shimodaira, 2002).

**Table 4:** Genome basics for twenty-three studied fungal genomes.

Assembly	No. of Contigs	Largest Contig (bp)	Total Length (Mb)	GC (%)	N50 (bp)	N75 (bp)	BUSCO Completeness (%)	Genes Predicted	Proteins Predicted
***Alectoria*** ***sarmentosa***	915	400,628	39.9	40.22	93,085	44,808	75.8	8440	8406
***Bacidia*** ***gigantensis***	24	3,530,911	33.1	44.67	**1,807,239**	**1,552,797**	**95.8**	8451	8400
***Bacidia rubella***	246	2,353,056	33.7	45.25	**1,771,855**	**1,480,693**	**97.9**	8773	8728
***Cladonia grayi***	414	958,967	34.6	44.44	243,412	104,892	**96.8**	9215	9168
***Cladonia*** ***macilenta***	240	2,265,542	37.1	44.68	**1,469,036**	**1,071,353**	**96.8**	8183	8135
***Cladonia*** ***metacorallifera***	30	2,400,105	36.6	44.91	**1,591,850**	**1,304,658**	**97.5**	8357	8313
***Cladonia*** ***rangiferina***	1008	751,829	35.6	45.46	273,041	142,056	**98.2**	9264	9218
***Cladonia*** ***uncialis***	2124	143,175	32.8	46.38	34,871	18,367	**91.7**	8706	8645
***Cyanodermella asteris***	37	3,440,352	28.6	53.29	**1,790,936**	**1,105,189**	**97.2**	7946	7851
***Dibaeis*** ***baeomyces***	1369	352,342	35.2	47.02	70,496	37,098	**97.9**	9799	9756
***Endocarpon*** ***pusillum***	908	803,103	37.1	46.00	178,225	78,254	**93.1**	8446	8392
***Evernia prunastri***	277	732,541	40.3	48.97	264,454	154,311	**97.9**	11,072	10,979
***Graphis scripta***	1453	383,549	36.2	46.66	78,723	38,837	88.6	9808	9744
***Gyalolechia flavorubescens***	36	2,816,824	34.4	41.89	**1,693,300**	**1,515,355**	**97.5**	8062	8008
***Lasallia*** ***hispanica***	1619	615,827	41.2	51.28	145,035	51,438	**97.9**	8218	8162
***Lasallia*** ***pustulata***	43	3,307,933	32.9	51.67	**1,808,250**	**1,551,388**	**98.2**	6973	6936
***Letharia*** ***columbiana***	161	2,188,364	52.2	39.57	666,803	377,091	**92.0**	9966	9890
***Letharia lupina***	31	3,031,725	49.2	38.73	**2,098,233**	**1,574,492**	**96.2**	9266	9206
***Pseudevernia furfuracea***	46	3,053,396	37.7	47.86	**1,178,799**	859,355	**97.2**	9148	9082
***Ramalina*** ***intermedia***	196	898,913	26.2	51.89	273,318	142,876	**97.5**	7405	7355
***Ramalina*** ***peruviana***	1657	694,821	26.9	50.75	40,431	15,829	90.0	6756	6706
***Usnea*** ***hakonensis***	879	624,317	41.1	45.57	166,123	71,534	**96.5**	10,700	10,641
***Xanthoria*** ***elegans***	261	990,773	44.3	40.70	385,707	188,494	**98.2**	9033	8911

## Data Availability

The data supporting the findings of this study are available open access in figshare at http://doi.org/10.6084/m9.figshare.19487837. These include gff3 files and protein sequences from twenty-two re-annotated genomes downloaded from the NCBI. In addition, the alignment used for the PKS tree calculation is deposited. The de novo sequenced genome of *Bacidia rubella* is available under the NCBI BioProject accession number PRJNA821848 and on the Sequence Read Archive (SRA).
